# Mesenchymal stem cells promote human melanocytes proliferation and resistance to apoptosis through PTEN pathway in vitiligo

**DOI:** 10.1186/s13287-019-1543-z

**Published:** 2020-01-15

**Authors:** Lifei Zhu, Xi Lin, Lin Zhi, Yushan Fang, Keming Lin, Kai Li, Liangcai Wu

**Affiliations:** 1 Department of Dermatology, The Sixth Affiliated Hospital of Sun Yat-sen University, Guangzhou, 510655 China; 2 Pharmacology Department of Basic Medical Sciences School of Medicine, Jinan University, Guangzhou, 510632 China; 3 Guangdong Provincial Key Laboratory of Colorectal and Pelvic Floor Disease, The Sixth Affiliated Hospital of Sun Yat-sen University, Guangzhou, 510655 China; 4 Guangdong Research Institute of Gastroenterology, The Sixth Affiliated Hospital of Sun Yat-sen University, Guangzhou, 510655 China

**Keywords:** PTEN, MSCs, Oxidative stress, Vitiligo

## Abstract

**Background:**

Vitiligo is an acquired chronic and recurrent skin disease that causes a depigmentation disorder, resulting in selective destruction of melanocytes (MC). However, the mechanism that leads to melanocyte dysfunction and death remains unclear.

**Methods:**

We performed RNA sequencing, immunohistochemistry, and immunoblotting to characterize the patterns of phosphatase and tensin homolog (PTEN)/phosphatidylinositol 3 kinase (PI3K)/protein kinase B (AKT) pathway activation in vitiligo. We also cocultured primary melanocytes with mesenchymal stem cells (MSCs) in a Transwell system to explore how MSCs inhibit the PTEN/PI3K/AKT pathway in melanocytes.

**Results:**

We identified that vitiligo normal-lesional junction skin presented with high expression of PTEN, which led to the inhibition of AKT phosphorylation (p-AKT) at S^-473^. Furthermore, PTEN overexpression led to oxidative stress-induced apoptosis in melanocytes. Coculturing with MSCs enhanced the cell proliferation of human melanocytes and repressed PTEN expression, which inhibited oxidative stress-induced apoptosis.

**Conclusion:**

We report that vitiligo patients present with high PTEN expression, which may play a role in the impairment of melanocytes. Furthermore, our study provides evidence that MSCs target the PTEN/PI3K/AKT pathway to regulate cell proliferation and apoptosis in human melanocytes, indicating that MSCs may serve as a promising therapy for vitiligo.

## Introduction

Vitiligo is a disfiguring disorder characterized by depigmented patches in the skin that are caused by deficiency or dysfunction in melanocytes [1, 2]. Approximately 1% of the population in the world are affected by vitiligo, and there is no cure approved by the US Food and Drug Administration [3]. Research has shown that vitiligo is a multifactorial disease that involves both genetic and environmental factors. Environmental factors, such as UV light, accelerate the generation of intracellular reactive oxygen species (ROS) and hydrogen peroxide and the initiation of melanogenesis, which liberates hydrogen peroxide [4]. Cellular stress induces ROS and activates the unfolded protein response (UPR), which exacerbates vitiligo. This response in turn leads to the production of inflammatory cytokines (interleukin (IL)-6 and IL-8) by melanocytes, which can initiate a signaling cascade and antagonize the suppressive function of regulatory T cells [5]. However, due to their specialized function of producing melanin, an energy-expensive process, epidermal melanocytes are particularly vulnerable to oxidative stress [6]. There is increasing evidence showing that excessive ROS production induced by sun exposure and inflammation can impair melanocyte homeostasis, leading to functional deficiency and death in the cells or, in the worst case, leading to malignant transformation [6–8]. In practice, some treatments can restore pigment, but many white patches and some other stable types of vitiligo react poorly to these treatments, with melanocytes playing a critical role in the outcome of the treatments [9]. Research shows that depigmented skin presents with high levels of epidermal H_2_O_2_ and peroxynitrite, and the key to restoring pigment in depigmented skin is reducing the H_2_O_2_ level [10, 11].

Studies have demonstrated that MSCs suppress immune reactions and reduce inflammatory cytokine levels [12, 13]. MSCs are characterized as having multipotent differentiation potential and limited immunogenicity and are poorly recognized by HLA-incompatible hosts. Based on these properties, MSCs can serve as an effective tool for tissue repair [14]. Significantly, MSCs have been shown to secrete several trophic factors, such as paracrine molecules, that promote the nutrition effect on neighboring cells, enhancing proliferation and functions during in vitro culture [13, 15]. The interactions of cells with other cell types and with their surrounding environment are critical for tissue development, maintenance, repair, and homeostasis [16]. Additionally, in vitro and preclinical trials have proven that MSC therapy suppresses oxidative stress. MSCs have shown antioxidant effects on a mouse model of multiple sclerosis by inducing high expression of antioxidant enzymes such as superoxide dismutase, catalase, and poly (ADP-ribose) polymerase-1 in response to treatment with an intravenous injection of MSCs [17]. Moreover, the transplantation of human amniotic MSCs into transgenic mice produces decreased levels of lipid peroxidation and oxidative stress and increased levels of antioxidant enzymes [18]. Crosstalk between keratinocytes (KE) and melanocytes has been well studied. The production of numerous synergistic mitogens by keratinocytes and direct cell-to-cell contact promote the proliferation of melanocytes ex vivo [19]. However, whether MSC-secreted mediators affect the survival and function of human melanocytes is still unclear.

PTEN is regarded as one of the most important tumor suppressors and is characterized as inhibiting the PI3K oncogenic pathway [20, 21]. The important functions of PTEN in cell growth, proliferation, and migration suppression; apoptosis promotion; DNA damage repair; tumor suppression; and metabolism have been well documented [20]. PTEN regulates cell growth by causing cell cycle arrest and negatively influencing cell survival, largely due to its cytoplasmic activity against the PTEN/PI3K/AKT pathway [22]. The PTEN/PI3K/AKT pathway also plays an important role in regulating the immune system. Increasingly, studies have focused on the decrease in PTEN expression in melanoma and other types of cancer [23, 24]; however, the function of PTEN in vitiligo is still unclear. The objective of this study was to identify some of the signaling networks involved in the initiation and progression of vitiligo and target these pathways therapeutically to establish a vitiligo treatment strategy.

## Methods

### Skin tissue samples

Skin specimens for RNA sequencing (*n* = 3), immunohistochemistry (*n* = 3), and western blotting (*n* = 11) were obtained at the dermatology outpatient department from subjects diagnosed with active vitiligo (VIDA ≥ 2) who were not receiving any other treatments. The normal, junction, and repigmented skin specimens that we used as controls were obtained from the same vitiligo patients.

### Melanocytes, MSCs, keratinocytes, cell line culturing, and a coculture assay

Pure primary melanocytes were generated from foreskin obtained from male circumcision patients (*n* = 40) aged 20–25 years old. The foreskin specimens were washed with 1% penicillin-streptomycin antibiotic-supplemented DPBS and digested with a 0.25% Dispase II solution at 4 °C for 16–18 h. The epidermis and dermis were separated, and the epidermis was digested with 0.25% trypsin for 10–15 min and incubated with M254 medium (Gibco, Grand Island, NY) containing 10% fetal bovine serum (Gibco) and 1% Human Melanocyte Growth Supplement-2 (Gibco) at 37 °C in a 5% CO_2_ atmosphere [25]. Keratinocytes were obtained with the same protocol and incubated with KSFM (Gibco) and serum-free medium. The primary melanocytes we used for each experiment were second or third passage cells and exhibited an elongated, multipolar morphology. Melanocytes were cultured in M254 complete medium and had a doubling time of 10–14 days. The melanoma cell line SK-Mel-110 was purchased from ATCC and cultured in RPMI 1640 medium (Gibco) supplemented with 10% fetal bovine serum. MSCs were a gift from the School of Life Sciences, Sun Yat-sen University, and these cells were maintained in DMEM (Gibco) supplemented with 10% fetal bovine serum. The MSCs we used had a fusiform shape, were arranged in bundles or whorls, became confluent with a doubling time of 3–4 days, and were passaged 2–3 times. A coculture assay was performed with a Transwell system with melanocytes seeded in the bottom chamber, MSCs, and keratinocytes seeded in the upper chamber at a ratio of 2:1, 1:1, or 1:2.

### Antibodies and reagents

Antibodies against PTEN and nuclear factor, erythroid 2-like 2 (Nrf2), were obtained from Abcam, and antibodies against p-S473-AKT and AKT were purchased from Cell Signaling Technology. Secondary antibodies for western blotting and immunofluorescence were purchased from Invitrogen.

### RNA sequencing

Skin specimens were isolated from vitiligo patients (*n* = 3). Melanocytes were cocultured with MSCs for 12 h as the treatment group (Tre) and compared with noncocultured melanocytes in the control group (Con). The melanocytes cocultured with MSCs versus noncocultured melanocytes were collected for 3 independent experiments. Both tissue specimens and cells were stored in liquid nitrogen and evaluated by RNA sequencing. RNA was extracted using a RNeasy kit (QIAGEN). The isolated mRNA was reverse transcribed and amplified by PCR using universal primers. Gene expression intensity data were obtained by denaturing and hybridizing the PCR products to Illumina arrays using Illumina GenomeStudio software. We obtained gene-based fragments per kilobase of exon per million fragments mapped (FPKM) values for all samples by using Cufflinks (version 2.1.1). The expression values for all probes and samples were log (base 2) transformed. Differential expression analysis was performed for each Illumina array probe. Significant enrichment in functionally related categories was examined by Kyoto Encyclopedia of Genes and Genomes (KEGG) pathway analysis for genes passing specific distinguishing criteria.

### Gene set enrichment analysis (GSEA)

We ranked the transcriptomic data for 17,138 genes by their associations with the junction (*n* = 3) and normal (*n* = 3) groups using the signal-to-noise measure in a GSEA. *P* value < 0.05 was considered statistically significant.

### Immunohistochemical, immunofluorescence, and dopa staining

Skin tissue samples were fixed with 4% polyformaldehyde overnight and then dehydrated in an ethanol gradient. Then, antibodies specific for PTEN, p-S473-AKT, and AKT were utilized at a dilution of 1:100. Melanocytes were cocultured with MSCs for 12 h, the antibodies against PTEN were diluted at a ratio of 1:80, and incubated with the MSCs for 12–16 h at 4 °C, and a secondary antibody was diluted at a ratio of 1:100. A 2 mg/ml dopa phosphate-buffered solution was used for dopa staining for 1 h at 37 °C.

### PTEN plasmid transfection

Second- or third-generation primary melanocytes were seeded in 6-well plates and incubated overnight, after which time they were transfected with a PTEN plasmid (mixed with Lipo 3000 in a ratio of 1:1). After 6 h, new medium was added. Subsequent treatments (coculture assay) were performed after 30 h.

### Western blot analysis

Specimens were obtained from vitiligo patients and analyzed in a lysis buffer supplemented with 1% protease inhibitor (Sigma) after being gently washed twice with DPBS, and similar procedures were performed with cocultured primary melanocytes and SK-Mel-110 cells. Lysate supernatant was collected after centrifugation at 13,000*g* for 15 min. Protein (20 μg) was loaded onto 4–10% SDS-PAGE minigels and incubated with primary antibodies specific for PTEN (1:1000), p-S473-AKT (1:1000), AKT (1:1000), and Nrf2 (1:1000) for 12–16 h at 4 °C with constant shaking, followed by incubation with a secondary antibody for 1 h at room temperature. The quantification of bands was performed with Image-Pro Plus, and the results were normalized to those for the control GAPDH.

### Cell counting kit 8 (CCK-8) assay

Second or third passage primary melanocytes from 10 different individuals aged 20–25 years old were seeded in a 12-well plate and cocultured with MSCs at a ratio of 2:1, 1:1, or 1:2. CCK-8 (30 μl, Tokyo, Japan) was added into 300 μl cell medium 254 and incubated at 37 °C for 180 min. The optical density value (OD value) was assessed by using a spectrophotometer reader from Thermo Fisher Scientific (Waltham, MA, USA) at an absorbance of 450 nm.

### Flow cytometry

Second or third passage primary melanocytes were seeded in 12-well plates at a density of 30,000 cells/cm^2^, treated with 1 mM H_2_O_2_ for 30 min, changed to complete medium, and then cocultured in a Transwell system containing the same density of MSCs and keratinocytes. The samples were then incubated for 12 h and evaluated with an apoptosis kit (BestBio China). Apoptosis was then detected by flow cytometry (Beckman Coulter). The same protocol was used for the melanoma cell line SK-Mel-110.

### Data analysis

All data were obtained from two or three independent experiments and are shown as the mean ± SD. SPSS 20.0 was used to perform Student’s *t* test, where ns represents no statistical significance, * represents *P* value < 0.05, ** represents *P* value < 0.01, *** represents *P* value < 0.001, and **** represents *P* value < 0.0001. Graphs were drawn with GraphPad Prism software, and *P* values less than 0.05 were considered statistically significant.

## Results

### RNA sequencing and functional clustering analysis of active vitiligo specimens

**Fig. 1:**
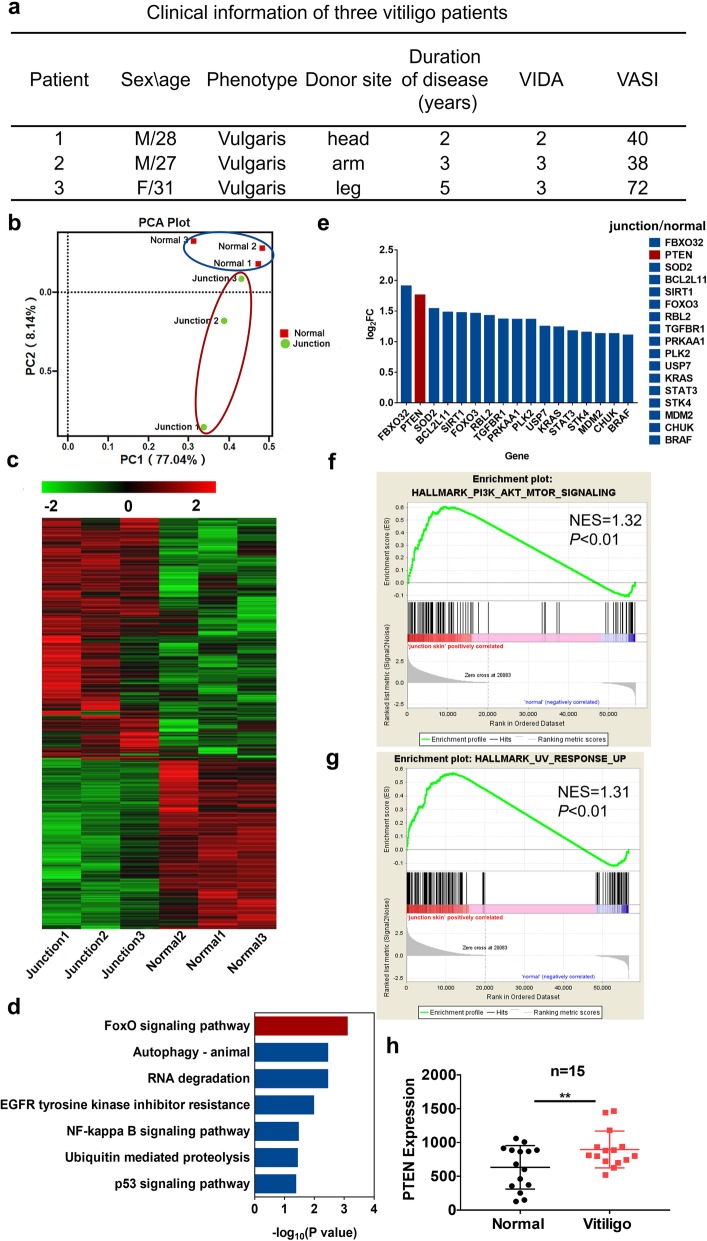
Vitiligo patients presented with high PTEN expression. **a** Clinical data of vitiligo patients (*n* = 3). **b** Principal component analysis of vitiligo skin samples. **c** Heatmap of vitiligo skin samples. The color scale indicates the log2 ratio of the normalized hybridization signal intensities of the regulated genes with a range of − 2.0 to + 2.0. **d**, **e** Regulated genes enriched in the KEGG database. Enrichment pathway analysis was performed in aggregated gene-base, and functional categories were set with a gene count log10 (*P* value< 0.05). The top upregulated pathway was selected and analyzed by log_2_FC. **f** to **g** GSEA graph of the upregulated PI3K/AKT/mTOR pathway and UV response in the vitiligo skin samples. *P* value < 0.01. **h** Transcriptomic data analysis of vitiligo lesion skin and non-lesion skin in GEO DataSets. PTEN expression in this study (series: GSE75819, *n* = 30). ** *P* value < 0.01

Targeted therapeutics in the treatment of vitiligo would be a substantial advance. In order to explore the key pathways that drive vitiligo pathogenesis, we used RNA sequencing to capture the differences in gene expression between vitiligo normal-lesional junction skin and normal skin from the same vitiligo patient. Patients were first diagnosed as having active segmental vitiligo without any other related disease and did not receive any treatment or medication, and they were then evaluated to determine Vitiligo Disease Activity (VIDA) and Vitiligo Area Scoring Index (VASI) scores. The patients with a VIDA score greater than or equal to 2 points were recruited, and the clinical history data of three vitiligo patients are shown in Fig. 1a. Our RNA sequencing analysis showed that the clustering analysis distinguished the vitiligo samples from the normal samples, with PC1 and PC2 accounting for 71.04% and 8.14% of the variation, respectively (Fig. 1b). The regulated genes (2063) were aggregated into a heatmap, revealing that compared to the normal samples, the vitiligo junction samples had a unique gene expression profile with 1217 genes exhibiting increased expression and 846 genes exhibiting decreased expression (Fig. 1c). Next, we examined the expression of all regulated genes using the KEGG database. The enrichment analysis showed that the FoxO signaling pathway was significantly upregulated and ranked as the top pathway among all enriched pathways (*P* < 0.001) (Fig. 1d). FoxOs are implicated in a broad range of cellular functions, including cell differentiation, cell proliferation, DNA damage, apoptosis, and repair, and act as mediators of oxidative stress [26, 27]. Afterwards, we analyzed all genes within the FoxO signaling pathway and observed that PTEN expression was upregulated by 3.4 folds (*P* < 0.0001) (Fig. 1e). To further investigate the regulation of the activation of downstream elements by PTEN, we performed GSEA, and the data showed that the PI3K/AKT/mTOR signaling pathway was significantly activated, as was the UV response pathway (Fig. 1f, g). Indeed, the PI3K/AKT/mTOR signaling pathway is the main downstream target of PTEN and is negatively regulated by PTEN. To confirm the regulatory function of PTEN, we evaluated data from Gene Expression Omnibus (GEO) DataSets. Researches by Singh et al. (series: GSE75819) and Regazzetti et al. (series: GSE65127) on 15 and 10 samples, respectively, also showed that PTEN expression in the lesional or peri-lesional skin was significantly higher than that in the non-lesional or healthy skin (*P* value< 0.05) (Fig. 1h, s) [28, 29]. Overall, the gene expression of vitiligo patients was regulated, and KEGG analysis demonstrated that the PTEN/PI3K/AKT pathway was upregulated in vitiligo tissue samples, suggesting that PTEN might be a potential marker of the initiation of vitiligo.

### PTEN expression is elevated in human vitiligo

**Fig. 2:**
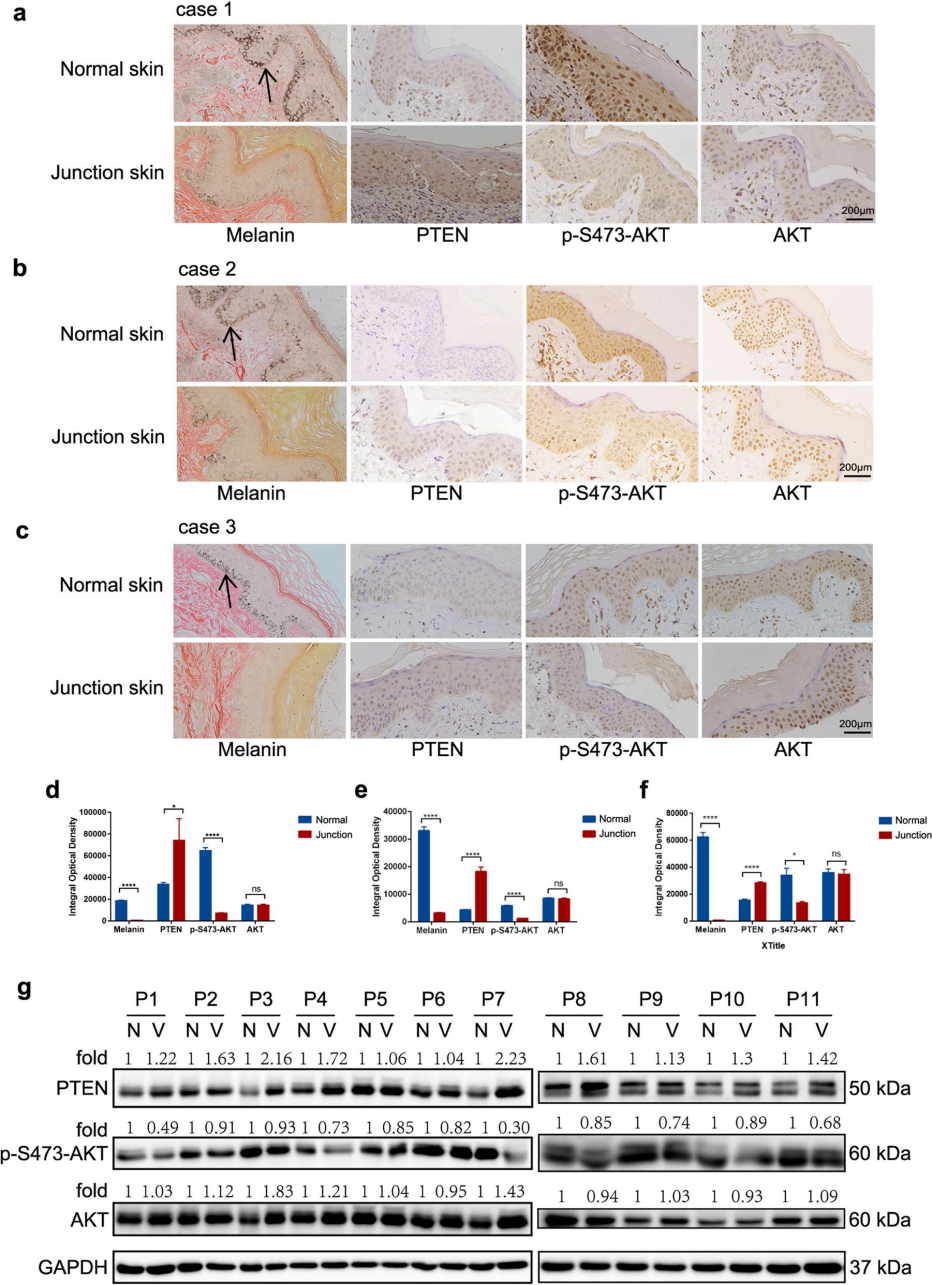
PTEN pathway was activated in active vitiligo lesions. **a**–**c** Immunohistochemical staining for melanin, PTEN, p-S473-AKT, and AKT; magnification, × 100; scale bar, 200 μm. **d**–**f** Quantification of immunohistochemistry data with Image-Pro Plus 6.0. **g** Immunoblotting analyses of PTEN, p-S473-AKT, and AKT. Qualification of immunoblotting bands was calculated by using Image-Pro Plus 6.0. Data analysis was performed by using SPSS 20.0. ns represents no statistical significance, * represents *P* value < 0.05, ** represents *P* value < 0.01, *** represents *P* value < 0.001, and **** represents *P* value < 0.0001

To verify the regulation of PTEN expression, we next detected the expression directly in vitiligo junction skin and normal skin. Argentaffin staining showed that normal skin had significant melanin enrichment, but vitiligo junction skin presented with little or no melanin. Immunohistochemistry analysis showed that PTEN expression in vitiligo skin was significantly higher than that in normal skin. Consequently, AKT phosphorylation was significantly lower in vitiligo skin than in normal skin, but there was no significant difference in AKT expression between the two groups (Fig. 2a–c). Quantification data are shown in Fig. 2d–f. To confirm the PTEN expression in vitiligo tissue, we performed immunoblotting with 11 specimens. The data showed that PTEN expression in vitiligo junction samples was 1.06- to 2.23-fold higher than that in the normal samples, while the phosphorylation of AKT at the 473 locus was downregulated. However, no significant change was observed in total AKT expression (Fig. 2g). Our finding shows that PTEN expression is increased in vitiligo junction skin, which subsequently inhibits the downstream AKT growth signaling pathway. This finding reveals that PTEN may play critical roles in the growth and survival of melanocytes, which might be crucial for vitiligo initiation and progression.

### MSCs downregulate PTEN expression in melanocytes in vitro

**Fig. 3:**
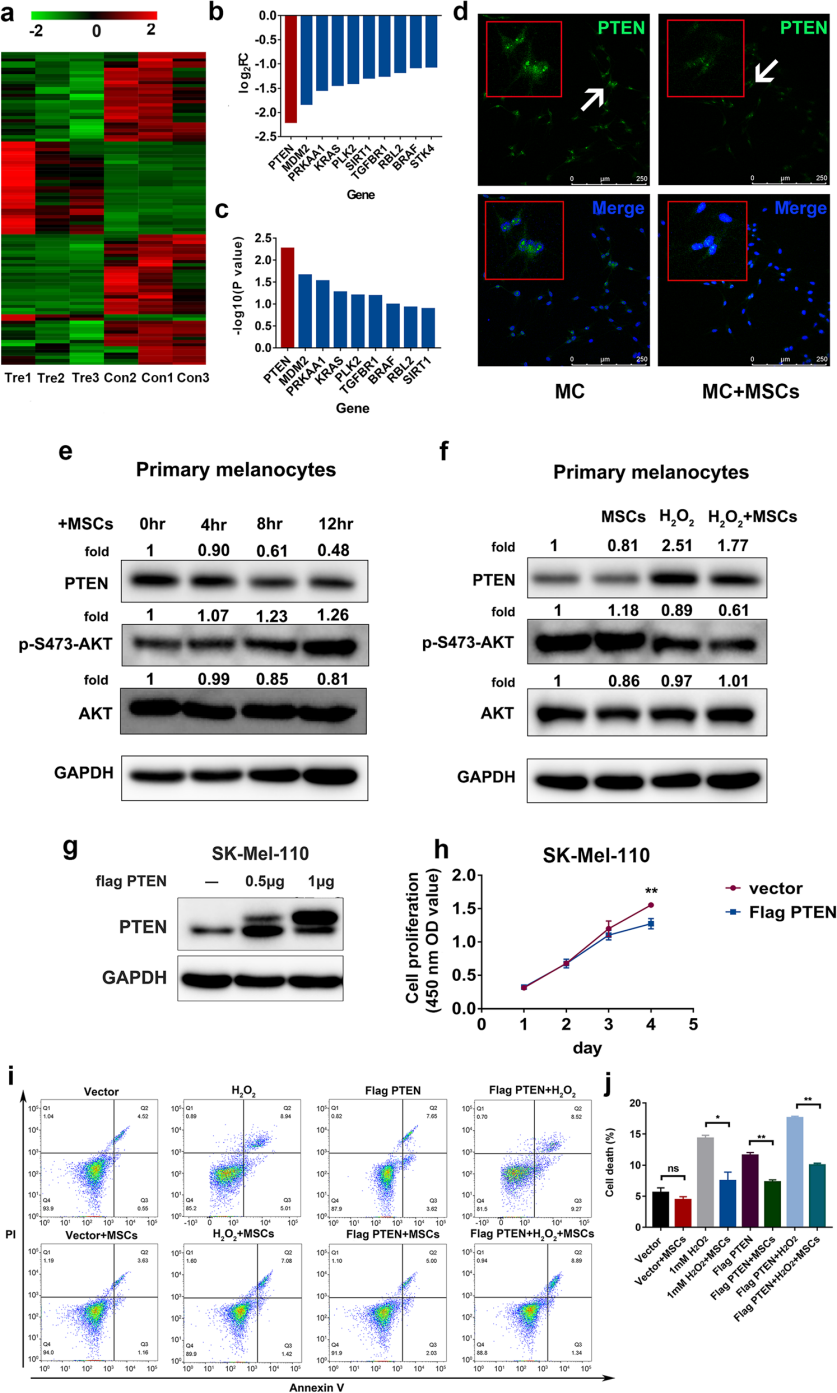
MSCs regulated PTEN expression in melanocytes. **a** Heatmap of cocultured primary melanocytes versus noncocultured melanocytes. The color scale indicates the log2 ratio of the normalized hybridization signal intensities of the regulated genes with a range of − 2.0 to + 2.0. **b** Log_2_FC of all genes in the FoxO pathway shown in Fig. 1. **c** Regulated genes calculated by log_10_ (*P* value). *P* value < 0.05. **d** Primary melanocytes cocultured with MSCs for 24 h and evaluated by immunofluorescence. Confocal microscopy was used to determine PTEN expression in both groups. **e** Primary melanocytes cocultured with MSCs for 0 h, 4 h, 8 h, or 12 h and analyzed by immunoblotting for PTEN, p-S473-AKT, and AKT. **f** Melanocytes pretreated with 1 mM H_2_O_2_ for 30 min and then cocultured with MSCs for 12 h. Immunoblotting analyses were performed for PTEN, p-S473-AKT, and AKT. **g** SK-Mel-110 cells treated with 0, 0.5 μg, or 1 μg flag PTEN plasmid and Lipo 3000 for 6 h and then evaluated. PTEN expression was measured by western blotting. **h** Cell proliferation of PTEN-overexpressing SK-Mel-110 cells evaluated by a cell counting kit 8 assay. **i**, **j** PTEN-overexpressing SK-Mel-110 cells treated with 1 mM H_2_O_2_ for 30 min. Cell death in all groups was measured by flow cytometry. Statistic data analyzed by Student’s *t* test. ns represents no statistical significance, * represents *P* value < 0.05, ** represents *P* value < 0.01

MSCs have been shown to produce and release growth factors, exosomes, and cytokines that affect cells in their vicinity, which are induced into different developmental pathways [30]. It has been reported that MSC-derived exosomes promote axonal growth by targeting PTEN/mTOR signals and function in wounding and repair through the PTEN pathway [31]. Recent advances have demonstrated that MSCs significantly increase the protein levels of the signaling growth factors PI3K, AKT, and mTOR and decrease the PTEN protein level. Furthermore, MSCs can inhibit PTEN expression, activate PI3K/AKT signaling, and lead to protection against oxidative stress-triggered cell death [32]. Therefore, we reasoned that MSCs might target some specific genes or signaling networks to function as a potential therapeutic approach for vitiligo. To investigate this, we performed RNA sequencing of both primary melanocytes cocultured with MSCs and noncocultured melanocytes. Comparison of the sequencing results of the two groups indicated that an array of genes was regulated by the MSCs. (Fig. 3a). Among these regulated genes, we found that the expression of PTEN was downregulated significantly (Fig. 3b, c). Next, we performed an immunofluorescence assay to determine how MSCs alter PTEN expression in melanocytes. Our data showed that the total amount of PTEN in the cocultured group was less than that in the noncocultured group (Fig. 3d). We cocultured melanocytes with MSCs for 0, 4, 8, or 12 h, and the results showed that PTEN expression was significantly downregulated in the MSC-cocultured group compared to the noncocultured group. Correspondingly, AKT phosphorylation was upregulated significantly after 12 h of coculture. Total AKT expression was not affected (Fig. 3e). ROS can suppress PTEN phosphatase activity through the formation of intramolecular disulfide. It has been reported that intracellular levels of H_2_O_2_ are significantly upregulated in the epidermis of vitiligo patients and high ROS levels can lead to constitutive oxidation and inactivation of the PTEN pool. Decreased PTEN lipid phosphatase activity is associated with PTEN overexpression [33]. Therefore, we further investigated how MSCs regulate PTEN expression in melanocytes under oxidative stress injury conditions. Our data showed that when primary melanocytes were treated with H_2_O_2_, PTEN expression was upregulated. This finding is consistent with the vitiligo skin RNA sequencing, immunohistochemical staining, and immunoblotting results described above, suggesting that the disease state of melanocytes was successfully mimicked. Under this condition, coculturing melanocytes with MSCs could downregulate PTEN expression and consequently lead to AKT phosphorylation (Fig. 3f), which again confirmed our previous findings. Additionally, overexpression of PTEN in Sk-Mel-110 cells also inhibited cell proliferation (Fig. 3g, h). We further tested how PTEN affects cell survival, and the data showed that Sk-Mel-11 cells with overexpression of PTEN were more likely to be damaged regardless of whether H_2_O_2_ was present. MSCs could prevent melanoma cells from dying due to PTEN overexpression and oxidative stress injury induced by H_2_O_2_ (Fig. 3i, j). Taken together, the evidence shows that MSCs can regulate PTEN expression in melanocytes and it might subsequently decrease the impairment caused by PTEN overexpression and oxidative stress induced by H_2_O_2_. Our findings imply that MSCs may target PTEN to function as a promising treatment for vitiligo.

### MSCs promote cell proliferation and reduce oxidative stress toxicity in primary melanocytes

**Fig. 4:**
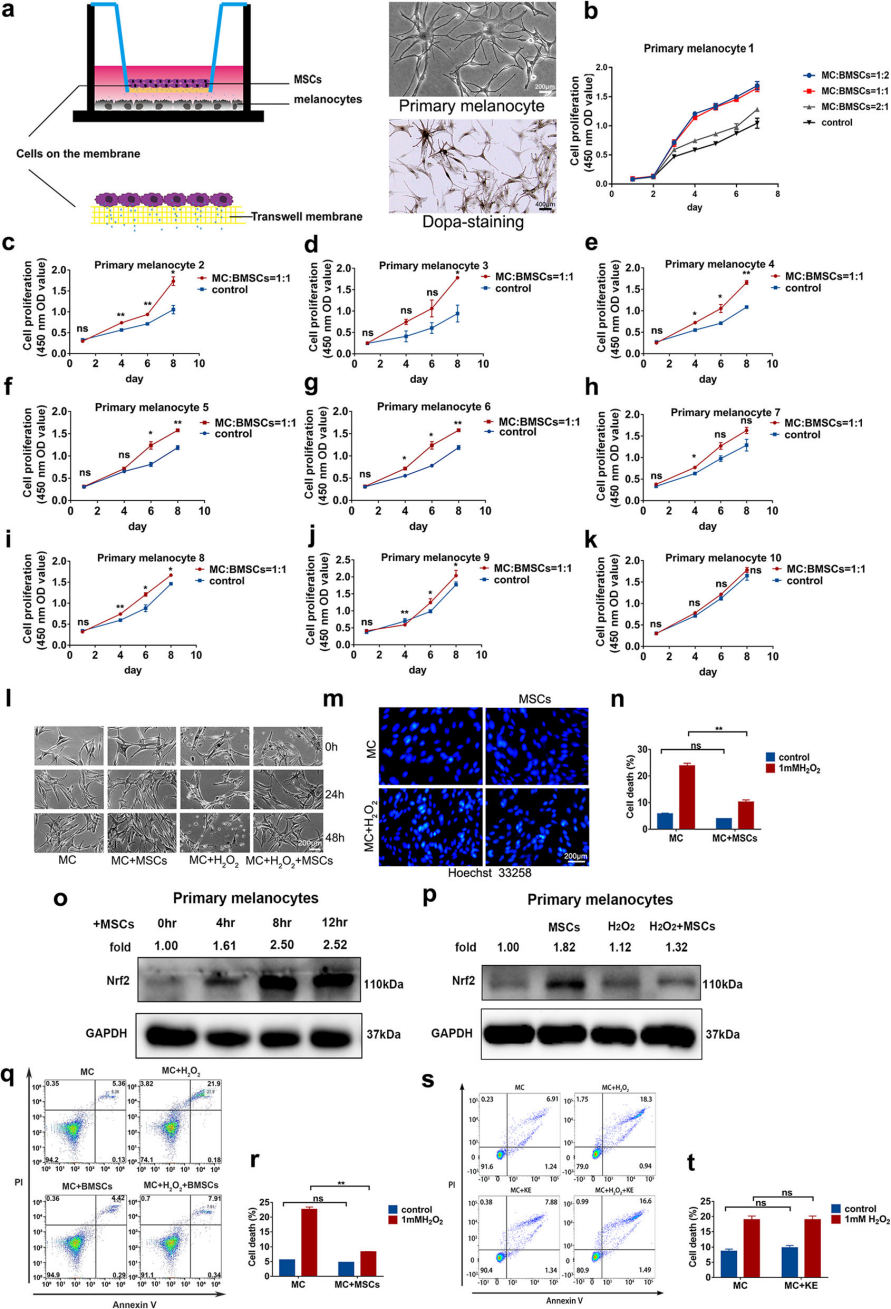
MSCs enhanced the cell proliferation of melanocytes and rescued melanocytes from oxidative stress. **a** Schematic diagrams of the Transwell coculture system, phase-contrast diagram and dopa staining of melanocytes; magnification, × 100; scale bar, 200 μm. **b**–**k** Cell proliferation of 10 primary melanocyte pools. Statistical data were analyzed by Student’s *t* test, and a *P* value < 0.05 was considered significant. **l** Phase-contrast microscopy analysis of primary melanocyte cell proliferation after treatment with 1 mM H_2_O_2_ for 30 min and coculture with MSCs for 24 h or 48 h; magnification, × 100; scale bar, 200 μm. **m** Hoechst staining of melanocytes; magnification, × 100; scale bar, 200 μm. **n** Statistical analysis of Hoechst staining with SPSS 20.0. **o** Primary melanocytes cocultured with MSCs for 0 h, 4 h, 8 h, or 12 h and analyzed by immunoblotting for Nrf2. **p** Melanocytes treated as described in **l**. Immunoblotting was used to evaluate Nrf2 expression. **q**, **r** Melanocytes treated as described in **l**. Flow cytometry was used to detect the cell death rate of each group. **s**, **t** Melanocytes pretreated with 1 mM H_2_O_2_ for 30 min and then cocultured with keratinocytes for 12 h, flow cytometry assay was used to detect the apoptosis rate. ns represents no statistical significance, and ** represents *P* value < 0.01

To assess the functional contributions of MSCs to primary melanocytes, we cocultured MSCs with 10 different melanocyte pools generated from circumcisions performed on healthy individuals aged 20–25 years old. Primary melanocytes that underwent dopa staining and were observed by phase-contrast microscopy exhibited an elongated bipolar or multipolar morphology. We next seeded MSCs and primary melanocytes in the top and bottom layers, respectively, of a Transwell system at a ratio of 2:1, 1:1 or 1:2. The schematic diagrams of the Transwell coculture system are shown in Fig. 4a. Our data showed that the ratios of 1:1 and 1:2 had stronger effects than the 2:1 ratio on the improvement of melanocytes. The Transwell coculture assay showed significant increases in cell proliferation in 8 of the 10 primary melanocyte pools (Fig. 4b–k). Therefore, these results show that MSCs promote the proliferation of melanocytes in a coculture system.

With phase-contrast microscopy, we found that MSCs improved the elongation and polar morphology of melanocytes in the coculture system. When pretreated with 1 mM H_2_O_2_ for 30 min, the primary melanocytes were slightly rounded. Afterwards, we placed them in a coculture group or solo group, and the coculture group presented a better shape and no cells in suspension, while the solo group included rounded cells in suspension (Fig. 4l). Hoechst staining showed that more nuclei were concentrated in the solo group (Fig. 4m, n). Furthermore, we cocultured melanocytes with MSCs for 0, 4, 8, or 12 h, and the results showed that Nrf2 expression was upregulated in the MSC-cocultured group compared to the noncocultured group (Fig. 4o). Under the condition of oxidative stress (H_2_O_2_)_,_ MSCs also can upregulate Nrf2 expression (Fig. 4p), suggesting that MSCs positively improved the antioxidant response of melanocytes. In addition, a flow cytometry assay showed that MSCs significantly decreased apoptosis in melanocytes (Fig. 4q, r). Epidermal melanocytes and keratinocytes form functional and structural unit in the skin, and keratinocytes are expected to exert important effects on melanocytes. It is reported that numerous factors, such as mitogens, produced in and released from keratinocytes are involved in regulating the proliferation, migration, and differentiation of epidermal melanocytes [19]. Therefore, we compared the effect of MSCs with keratinocytes to understand whether MSCs possess benefits towards melanocytes. Here, we performed flow cytometry assays to determine the difference in treatment efficacy between the MSCs and keratinocytes coculture methods. Our data showed that coculture with keratinocytes could not decrease H_2_O_2_-induced apoptosis (Fig. 4s, t). Collectively, our findings demonstrate that MSCs promote detoxification and cell proliferation in melanocytes, indicating that MSCs possess potential therapeutic properties in vitiligo.

## Discussion

**Fig. 5:**
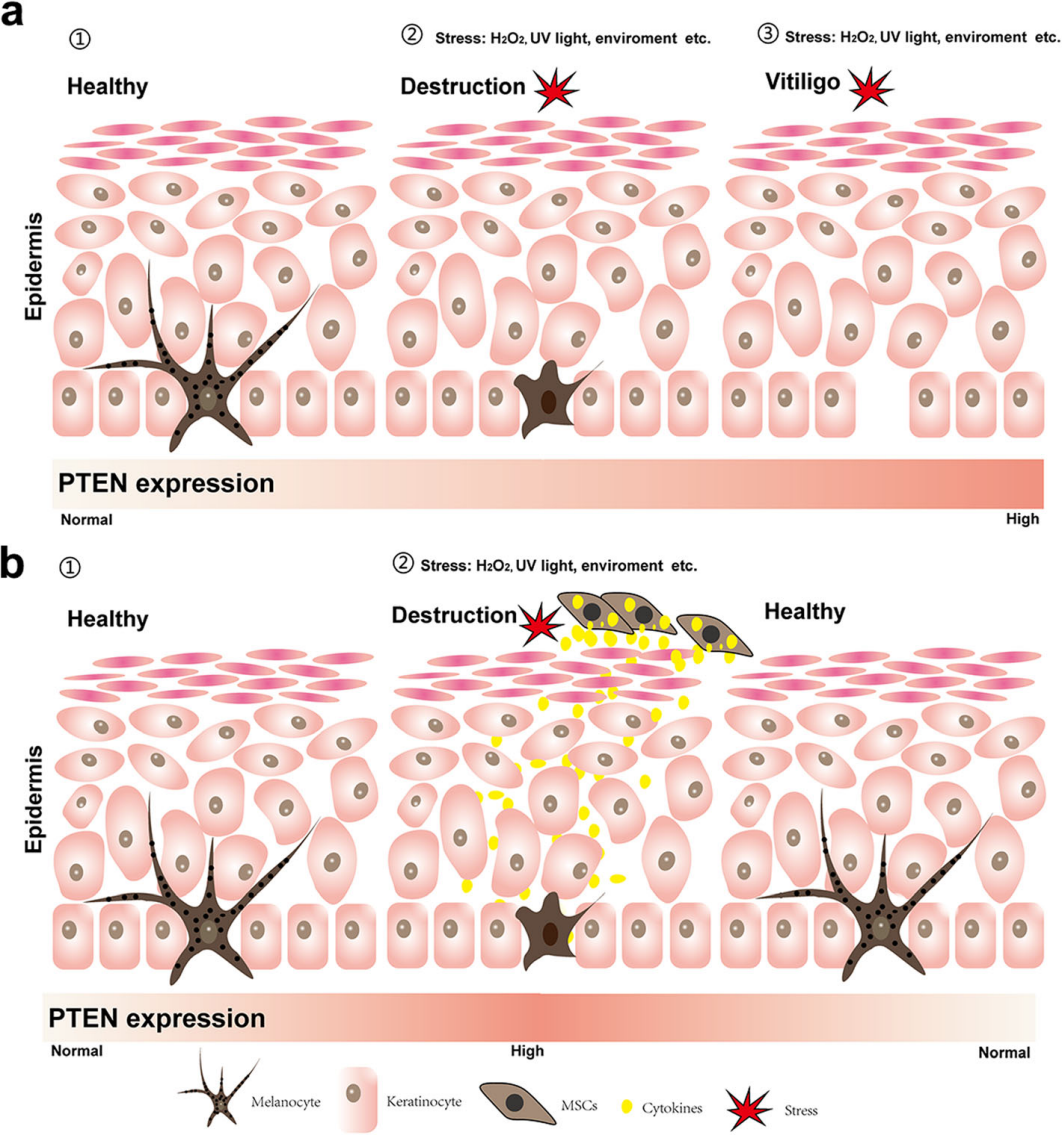
MSCs protect human melanocytes from dying by regulation of PTEN pathway. **a** High PTEN expression and oxidative stress damage melanocytes, leading to melanocyte death and eventually initiating vitiligo. **b** High PTEN expression and oxidative stress destroy melanocytes, while the presence of MSCs, which regulate PTEN expression and oxidative stress, rescues melanocytes from dying and restores their function of pigment production

As a classic tumor suppressor gene, PTEN mutation or deletion is involved in the development of both heritable and sporadic cancers. PTEN governs a variety of biological processes; therefore, the decrease of PTEN activity and levels will contribute to cancer susceptibility and favors tumor progression. Studies have proven that the PTEN mutation or deletion was observed in approximately 30% of human melanomas [34], yet the upregulated of PTEN expression is barely reported and the consequence of this type of regulation is still unknown. Our finding shows that vitiligo tissues exhibited elevated PTEN expression, which consequently led to reduced phosphorylation of AKT, revealing that vitiligo might be associated with PTEN pathway. Moreover, MSCs promote cell proliferation and reduce apoptosis of primary melanocytes in the coculture system, which may shed light on a new therapeutic treatment for vitiligo through restoration of PTEN tumor suppressor activity (Fig. 5a, b).

PTEN is associated with various cellular activities. PTEN decreases AKT signaling by dephosphorylating PIP3. PTEN produces the lipid second messenger, PIP3, to induce the transfer of AKT from the cytoplasm to the cell membrane, with Thr308 and Ser473 being subsequently phosphorylated by PDK1 and the mTORC2 complex, respectively. This process ultimately inhibits cell growth, cell cycle progression, cell survival, and proliferation and promotes apoptosis [35, 36]. A genetic study has shown that the rates of alterations in PTEN in melanoma cell lines, primary melanoma, and metastatic melanoma are 27.6%, 7.3%, and 15.2%, respectively. Additionally, a functional study has proven that progression in many melanoma cases is driven by the loss of PTEN expression and functions. Overall, PTEN is critical in regulating cell cycle progression and cell death in melanocytes, as well as melanoma tumorigenesis and metastasis [34, 37]. Our data shows that high PTEN expression induces melanocyte death and dysfunction, which might ultimately result in depigmentation and vitiligo. However, how PTEN affects gene transcription and activates the relevant pathway to promote the progression of vitiligo remains unclear. Why PTEN is highly expressed in vitiligo skin has never been examined, and it remains unknown whether PTEN expression is the outcome of any other genetic alterations or leads to a certain disease state. More studies need to be performed to understand the underlying mechanism.

The expression of immunosuppressive cytokines, including IL-6, IL-10, and VEGF, and programmed cell death 1 ligand (PD-L1) induced in a PI3K pathway-dependent manner is negatively regulated by PTEN, which supports the conclusion that PTEN may enhance host immune reactions by suppressing the expression of immunosuppressive cytokines and PD-L1 [23]. Consistently, our data showed that PTEN was highly expressed in vitiligo lesions, which might regulate the host immune response to participate in apoptosis induction in melanocytes and the cell-mediated killing of melanocytes and eventually result in depigmentation. However, more well-designed studies need to be performed to determine how PTEN expression regulates the immune response and vitiligo progression.

MSCs have been shown to secrete large quantities of cytokines that can regulate the microenvironment for tissue repair and improve cell proliferation through angiogenesis and the evasion of apoptosis in damaged cells [38, 39]. MSCs constitutively express superoxide dismutase, catalase, and glutathione peroxide to efficiently manage oxidative stress to resist oxidative stress-induced death [40]. Furthermore, MSCs can downregulate inflammation, oxidative stress, and levels of ROS and secondary products of lipid oxidation after transplantation [41]. Using CCK-8 and flow cytometry assays, we found that MSCs significantly improved cell proliferation and decreased apoptosis in melanocytes in a coculture system. We further verified that MSCs modulated PTEN expression in melanocytes in the presence of oxidative stress and subsequently increased AKT phosphorylation. Our findings imply that MSCs may modulate the cell proliferation of melanocytes by downregulating PTEN expression. While Kim et al. showed that human umbilical cord blood mesenchymal stem cells inhibit melanin synthesis by promoting proteasomal degradation of microphthalmia-associated transcription factor (Mitf) [42]. Here, we also evaluated Mitf expression and melanin content in melanocytes which were cocultured with MSCs, and the data showed that MSCs can downregulate Mitf expression and decrease melanin content (Additional file 1: Figure S2-S4). These findings suggested that mesenchymal stem cells have potential value of inhibiting melanogenesis in hyperpigmentation disease. Unlike focusing on how MSCs affects melanin production, our study showed that MSCs increase cell growth and resistance to apoptosis of melanocytes, and the data in vitro suggested that MSCs downregulated PTEN expression and depressed oxidative stress induced by H_2_O_2_. Thus, we reasoned that MSCs regulate PTEN expression and suppress oxidative stress by NRF2 pathway activation. More studies need to be done to investigate the mechanism inside. Taken together, it is suggested that mesenchymal stem cells may play a different role in maintaining cell proliferation, apoptosis, and melanin synthesis in melanocytes. Therefore, additional well-designed studies need to be performed to investigate the downstream mechanism.

Autologous melanocyte transplantation has served as an alternative surgical treatment for vitiligo lesions that are localized in poorly responsive areas [43], but how to maintain the biochemical and cellular stability of autologous melanocytes is still unclear. Due to the work of the Thomas PD team, it is known that melanocyte-intrinsic abnormalities may induce the whole inflammatory cascade [44]. It has been proven that melanocytes from vitiligo patients are more vulnerable to oxidative stress than those from healthy individuals and that these cells are harder to culture in vitro than those from unaffected controls [45]. In response to stressors, ROS are released as an outcome of surgical treatments, and no traditional surgical therapy has successfully altered the inflammatory environment that may cause death and dysfunction in melanocytes, which would lead to depigmentation occurring again. Considering the ability of MSCs to rescue melanocytes that we proved above, and the numerous functions that MSCs possess in inflammation and oxidative stress, here we propose to cotransplant autologous MSCs and melanocytes in a ratio of 1:1 into vitiligo patients according to our cocultured result, approximately 5 × 10^5^ melanocytes and MSCs per 1 cm^2^ of skin surface [46]. To further explore the clinical efficacy of our hypothesis, our team will work on evaluating the curative effect of the ratio and number of cotransplanting both cells into refractory vitiligo patients.

## Conclusion

In conclusion, we identified vitiligo lesions with high PTEN expression and decreased AKT phosphorylation, which might induce death in human melanocytes. Indeed, MSCs improved cell proliferation and suppressed apoptosis in melanocytes in a coculture system, downregulated PTEN expression, and consequently activated the PTEN/PI3K/AKT pathway. This prompted us to consider that MSCs might target PTEN to promote the proliferation of melanocytes and that cotransplanting MSCs with autologous melanocytes might serve as a promising durable therapeutic strategy for vitiligo.

## Supplementary information

**Additional file 1: Figure S1.** PTEN expression of healthy, lesional, non lesional and peri-lesional skin (n=10). The microarray data were obtained from GSE65127 in GEO. **Figure S2.** Primary melanocytes cocultured with MSCs for 0 hours, 4 hours, 8 hours or 12 hours and analyzed by immunoblotting for Mitf. The quantification of bands was performed with Image-Pro Plus, and the results were normalized to those for the control GAPDH. **Figure S3.** Primary melanocytes cocultured with MSCs for 48 hours and the cellular melanin was extracted by using melanin extraction buffer (1M NaOH containing 10% DMSO) at 100°C for 30 min. **Figure S4.** The cellular melanin content were determined by measuring the absorbance at 450 nm using the spectrophotometer reader. SPSS 20.0 was used to perform the Student’s t-test, *P* < 0.05 were considered statistical significance. * represents *P* value <0.05.

## Data Availability

Not applicable.

## Abbreviations

AKT: Protein kinase B
CCK-8: Cell counting kit 8
Con: Control group
GSEA: Gene set enrichment analysis
KE: Keratinocytes
KEGG: Kyoto Encyclopedia of Genes and Genomes
MC: Melanocytes
MSCs: Mesenchymal stem cells
Nrf2: Nuclear factor, erythroid 2 like 2
OD value: Optical density value
p-AKT: Phosphorylation of protein kinase B
PI3K: Phosphatidylinositol 3 kinase
PTEN: Phosphatase and tensin homolog
ROS: Reactive oxygen species
Tre: Treatment group
UPR: Unfolded protein response
VASI: Vitiligo Area Scoring Index
VIDA: Vitiligo Disease Activity
