# Supplementary material for: Mesenchymal stem cells promote human melanocytes proliferation and resistance to apoptosis through PTEN pathway in vitiligo
Source: Stem Cell Res Ther. 2020 Jan 15;11:26. doi: 10.1186/s13287-019-1543-z (PMC6961270; doi:10.1186/s13287-019-1543-z)
Supplement: Supplementary file 1 — Additional file 1: Figure S1. PTEN expression of healthy, lesional, non lesional and peri-lesional skin (n=10). The microarray data were obtained from GSE65127 in GEO. Figure S2. Primary melanocytes cocultured with MSCs for 0 hours, 4 hours, 8 hours or 12 hours and analyzed by immunoblotting for Mitf. The quantification of bands was performed with Image-Pro Plus, and the results were normalized to those for the control GAPDH. Figure S3. Primary melanocytes cocultured with MSCs for 48 hours and the cellular melanin was extracted by using melanin extraction buffer (1M NaOH containing 10% DMSO) at 100°C for 30 min. Figure S4. The cellular melanin content were determined by measuring the absorbance at 450 nm using the spectrophotometer reader. SPSS 20.0 was used to perform the Student’s t-test, P < 0.05 were considered statistical significance. * represents P value <0.05. [file 13287_2019_1543_MOESM1_ESM.docx]

**Additional file 1**

Figure S1


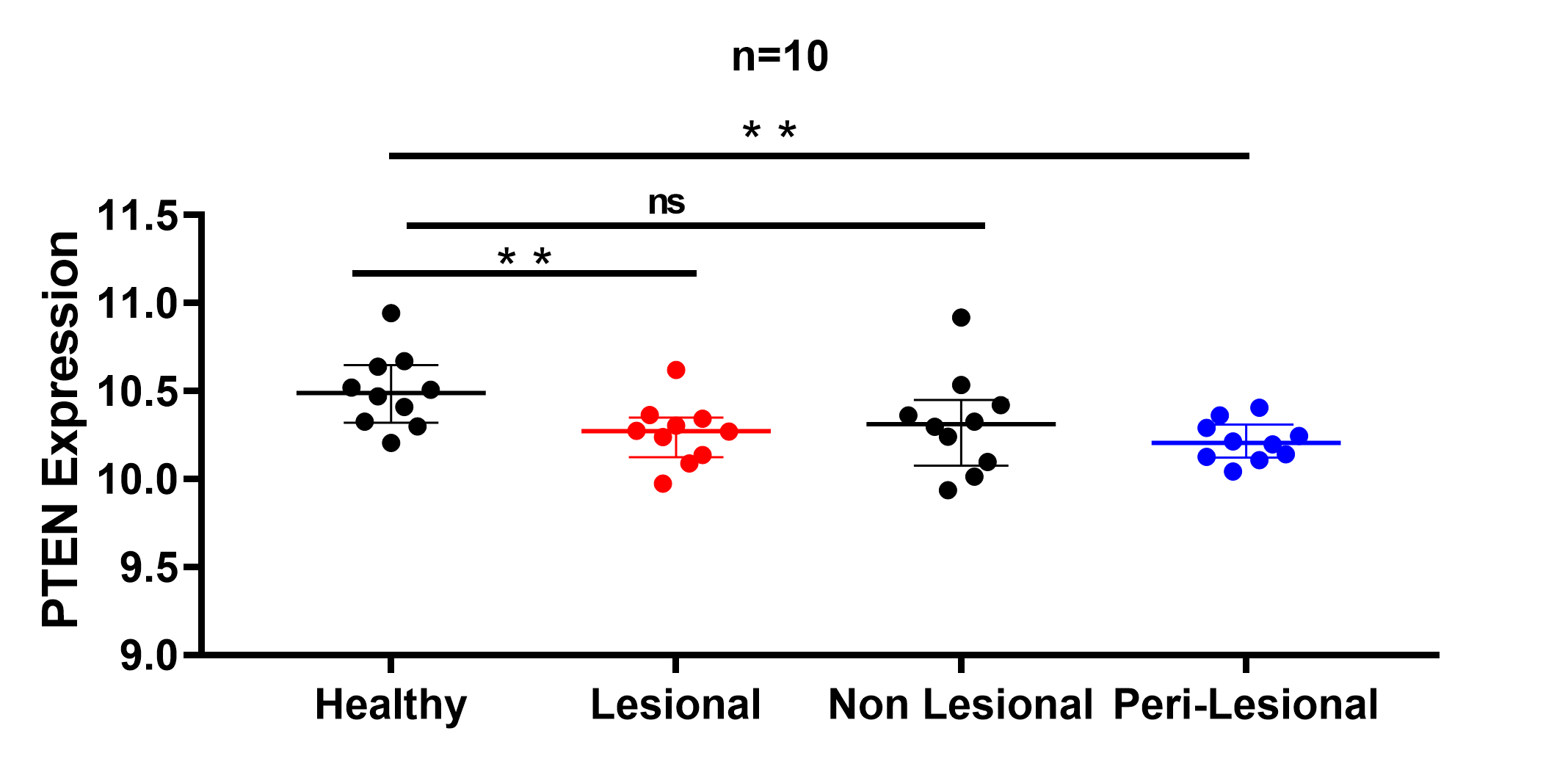


Figure S1: PTEN expression of healthy, lesional, non lesional and peri-lesional skin (n=10). The microarray data were obtained from GSE65127 in GEO.

Figure S2


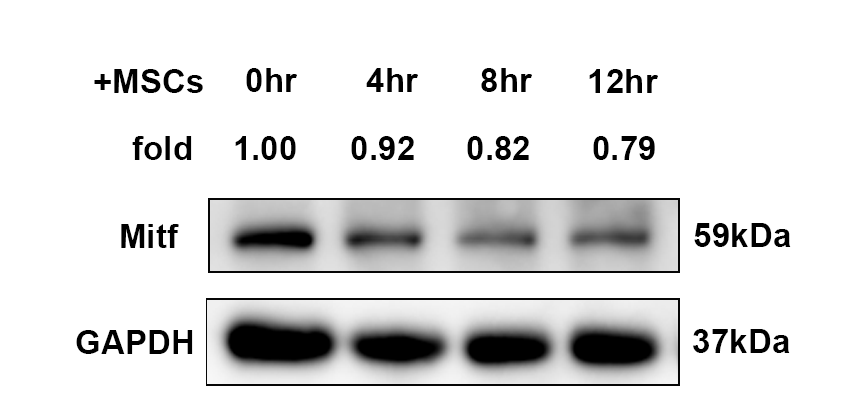


Figure S2: Primary melanocytes cocultured with MSCs for 0 hours, 4 hours, 8 hours or 12 hours and analyzed by immunoblotting for Mitf. The quantification of bands was performed with Image-Pro Plus, and the results were normalized to those for the control GAPDH.

Figure S3


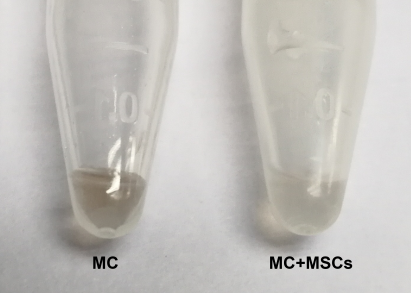


Figure S3: Primary melanocytes cocultured with MSCs for 48 hours and the cellular melanin was extracted by using melanin extraction buffer (1M NaOH containing 10% DMSO) at 100°C for 30 min.

Figure S4


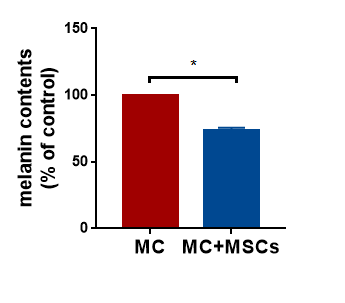


Figure S4: The cellular melanin content were determined by measuring the absorbance at 450 nm using the spectrophotometer reader. SPSS 20.0 was used to perform the Student’s t-test, *P* < 0.05 were considered statistical significance. * represents *P* value<0.05.
